# Embryotoxicity of pyrethroid insecticides and their mixtures in a human induced pluripotent stem cell-based model in vitro

**DOI:** 10.1007/s00204-026-04448-2

**Published:** 2026-05-20

**Authors:** Yanying Ma, Claudia Torero Gutierrez, Martin Scholze, Maria João Valente, Anne Marie Vinggaard

**Affiliations:** 1 National Food Institute, Technical University of Denmark, Kongens Lyngby, Denmark; 2 Centre for Pollution Research and Policy, Environmental Sciences Division, Brunel University London, Uxbridge, UK

**Keywords:** Pyrethroids, Mixture effects, Embryotoxicity, hiPSC-derived cardiomyocytes, PluriLum assay

## Abstract

Pyrethroid insecticides are used worldwide, yet their potential effects on early human development remain poorly understood. We applied the PluriLum assay, a human induced pluripotent stem cell-based 3D in vitro model, to evaluate the embryotoxicity of three commonly used pyrethroids (α-cypermethrin, deltamethrin, and etofenprox), and their shared metabolite 3-phenoxybenzoic acid (3-PBA). Embryoid bodies (EBs) were repeatedly exposed to these compounds throughout their differentiation into cardiomyocytes, and effects on cardiomyocyte beating, expression of the early cardiac marker *NKX2.5*, as well as their impact at a transcriptional level was assessed. The three pyrethroids were tested in combination at two different mixture compositions to investigate their potential for additivity. All three pyrethroids impaired cardiomyocyte differentiation at micromolar levels, with the following potency ranking: α-cypermethrin > etofenprox > deltamethrin. At higher concentrations, α-cypermethrin also reduced cardiomyocyte contractility. In contrast, 3-PBA showed no significant effects on neither differentiation nor contractile function. The pyrethroid mixtures followed the principle of concentration addition. An exploratory transcriptomic analysis revealed that α-cypermethrin and deltamethrin significantly altered the EBs gene expression profiles, affecting pathways related to ion channel activity, receptor signalling, and extracellular matrix organization. These findings suggest that exposure to pyrethroids may interfere with early human cardiac development through effects on multiple molecular targets. This study also highlights the value of the PluriLum assay as a human-relevant platform for assessing the embryotoxic potential of environmental chemicals.

**Supplementary Information:**

The online version contains supplementary material available at 10.1007/s00204-026-04448-2.

## Introduction

Pyrethroids are a large group of synthetic insecticides wildly used in agriculture and household products. Their chemical structures are related to pyrethrin, a naturally occurring insecticide in *Tanacetum cinerariifolium* flowers, but pyrethroids exhibit higher photostability and greater environmental stability as well as persistence (Marques et al. 2022). Commonly used pyrethroids such as allethrin, bifenthrin, cyfluthrin, cyhalothrin, cypermethrin, deltamethrin, etofenprox and flumethrin are rapidly metabolized in mammals, with metabolites excreted in urine and faeces within a few days after exposure (Kaneko 2010). In humans, metabolism primarily occurs in the liver, with elimination usually completed within several days (Viel et al. 2015).

Human exposure occurs mainly through dietary intake of pesticide residues, but indoor use of insecticides and environmental contamination from nearby agriculture areas are also important sources (Andersen et al. 2022a). In Denmark, monitoring data from 2012 to 2019 showed frequent detection of etofenprox in imported peaches and nectarines, deltamethrin in imported fruits and vegetables as well as domestically produced rye grain and wheat flour, and cypermethrin in both imported and domestically produced fruits and vegetables (Jensen et al. 2019). Based on this exposure relevance, we selected etofenprox, deltamethrin, and α-cypermethrin—the most biological active isomer of cypermethrin—for testing. In addition, 3-phenoxybenzoic acid (3-PBA), a urinary metabolite common to up to 20 pyrethroid insecticides, was included due to its widespread detection across European populations (Andersen et al. 2022b). Pyrethroid exposure is of particular concern during pregnancy as the lipophilic pyrethroids are able to easily cross the placenta barrier (Berton et al. 2014; Silver et al. 2015), resulting in foetal exposure. Moreover, several EU biomonitoring studies have detected pyrethroid metabolites in urine samples from pregnant women (Andersen et al. 2022b; Guimar et al. 2023).

Pyrethroids act primarily by targeting voltage-gated sodium channels in the central nervous system of exposed organisms (Soderlund 2010). Although mammals are less acutely sensitive to pyrethroids than insects (Hansen et al. 2017), prolonged or repeated exposure may lead to long-term human adverse health effects (Abreu-Villaça and Levin 2017; Hansen et al. 2017). Several epidemiological studies have linked prenatal pyrethroid exposure to impaired neurodevelopment in children (Rauh et al. 2006; Shelton et al. 2015; Eskenazi et al. 2018; Dalsager et al. 2019; Andersen et al. 2021). In contrast, much less is known about the effect of pyrethroids on cardiac development. Voltage-gated sodium channels are abundantly expressed in cardiomyocytes (Edokobi and Isom 2018; Vermij et al. 2020), raising concern that the heart may also be vulnerable to pyrethroids exposure. Experimental studies in rodents and zebrafish indicate that certain pyrethroids can disrupt cardiac function (Spencer et al. 2001; De la Cerda et al. 2002; Luo et al. 2019; Ghazouani et al. 2020; Guo et al. 2023). A cohort study of 2116 adults from the US further reported that elevated urinary 3-PBA levels were associated with increased risk of cardiovascular mortality over a 14-year follow-up period (Bao et al. 2020).

Voltage-gated sodium channels initiate rapid depolarization in cardiomyocytes, enabling propagation of the action potential and triggering calcium entry, which ultimately leads to myocardial contraction. Given this critical role of voltage-gated sodium channels in cardiomyocytes’ function, understanding pyrethroids effects on early cardiac development is essential. The heart is the first organ to form and function during embryogenesis, originating from mesodermal cells in the anterior lateral embryonic disc (Macgrogan et al. 2010). These cells are guided toward the cardiac lineage by signalling pathways such as Wnt inhibition and growth factors including bone morphogenetic proteins. As differentiation progresses, early cardiomyocytes begin to contract, driven by ion channel activity (Buijtendijk et al. 2020), including voltage-gated sodium channels, which mediate sodium influx essential for the excitability of heart cells and ensure the efficient conduction of electrical impulses needed for each heartbeat (Remme and Bezzina 2010; Abriel et al. 2016).

To investigate these processes in a human-relevant system, we employed embryoid bodies (EBs) derived from human-induced pluripotent stem cells (hiPSCs). EBs mimic early embryonic tissues and can differentiate into cell types from all three germ layers (Prochazkova et al. 2015), including cardiomyocytes. This platform enables evaluation of chemical embryotoxicity. To our knowledge, no previous study has investigated pyrethroid developmental cardiotoxicity in human-relevant in vitro models. Our study addresses this gap using a 3D hiPSC-derived cardiomyocyte differentiation model with the PluriBeat and PluriLum assays, which generate contracting and luminescence-emitting cardio-spheres by day 7 of differentiation, respectively (Lauschke et al. 2020, 2021; Treschow et al. 2024b). The PluriLum assay quantifies cardiac differentiation through a luciferase reporter gene transcribed in conjunction with the *NKX2.5* gene. Luminescence therefore provides an indirect and quantitative measure of cardiac differentiation. A reduction in luminescence indicates suppressed *NKX2.5* expression and potential inhibition of cardiomyocytes differentiation following pyrethroids exposure.

This study aimed to evaluate the effects and potential mechanism of selected pyrethroids and their common metabolite 3-PBA on cardiomyocyte differentiation. Additionally, we tested pyrethroid mixtures to assess potential interactions under combined exposure scenarios.

## Methods

### Chemicals and reagents

**Fig. 1:**
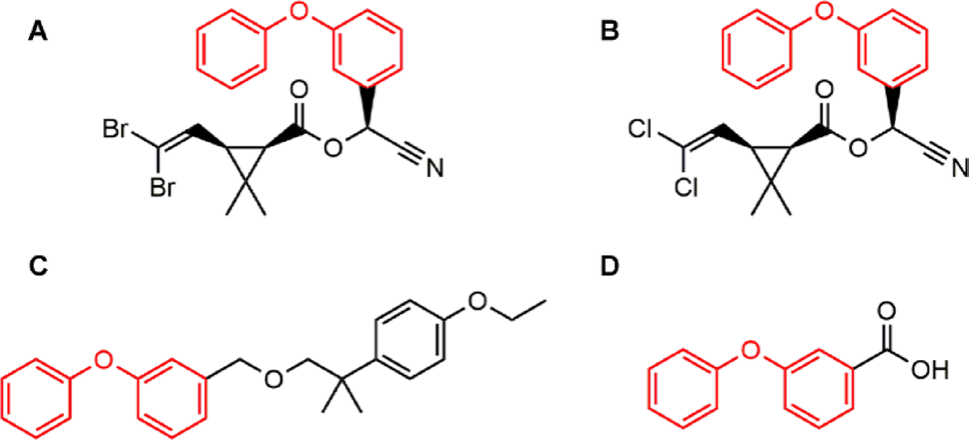
Chemical structures of (**A**) Deltamethrin, (**B**) α-Cypermethrin, (**C**) Etofenprox and their common metabolite (**D**) 3-Phenoxybenzoic acid. This figure was prepared using ChemDraw®, Revvity Signals

All reagents for the PluriLum assay were previously described (Treschow et al. 2024a). Briefly, mTeSR™ Plus medium was purchased from STEMCELL Technologies Inc. (Vancouver, Canada). hESC Qualified Matrigel® and ITS Premix Universal Culture Supplement (ITS) were acquired from Corning Inc. (NY, USA). Dulbecco’s Phosphate-Buffered Saline (DPBS), penicillin–streptomycin-glutamine (PSG), KnockOut™ DMEM medium, human fibroblast growth factor-basic (FGF2), activin A, 60 mm cell culture dishes, and 96-well Polystyrene Conical Bottom MicroWell™ plates were purchased from ThermoFisher Scientific Inc. (Massachusetts, USA). Human bone morphogenetic protein 4 (BMP4) and 4-(2-Methyl-4-pyridinyl)-N-[4-(3-pyridinyl)phenyl]benzeneacetamide (Wnt-C59) were purchased from Bio-Techne (Minnesota, USA). Sodium selenite, human transferrin, 2-phospho-L-ascorbic acid trisodium salt (Asc), papain, α-cypermethrin (CAS number 67375–30-8; > 98% purity), deltamethrin (CAS number 52918–63-5; > 98% purity), etofenprox (CAS number 80844–07-1; > 98% purity) and 3-phenoxybenzoic acid (3-PBA) (CAS number 3739–38-6; 98% purity) were acquired from Merck KGaA (Darmstadt, Germany). Y27632 dihydrochloride was purchased from Abcam Limited (Cambridge, UK), 6-(2-(4-(2,4-Dichlorophenyl)-5-(4-methyl-1H-imidazol-2-yl)-pyrimidin-2-ylamino)ethyl-amino)-nicotinonitrile (CHIR99021) was purchased from Axon Medchem BV (Groningen, the Netherlands). Chemical structures of the test compounds are given in Fig. 1.

### Cell culture maintenance

The BIONi010-C-*NKX2.5*-T2A-*Nluc*-44.37 cell line was used for this study. This cell line is a genetically modified version of the hiPSC BIONi010-C cell line established by Bioneer A/S (Hørsholm, Denmark), where a NanoLuc luciferase (*Nluc*) reporter gene was introduced in the locus *NKX2.5* (Lauschke et al. 2021), a key cardiac transcription factor expressed during early cardiomyocyte differentiation (Lints et al. 1993; Akazawa and Komuro 2005). The cells were cultured in mTeSR™ Plus medium, and on culture dishes coated with hESC Qualified Matrigel®. Cells were incubated at 37 °C and 5% CO_2_, in a humid environment. Culture medium was changed every other day and cells were passaged once a week using 0.02% EDTA in DPBS. Cells were kept in culture for 8 passages and were used between passage 38 and 44.

### Cardiomyocyte differentiation

Cardiomyocyte differentiation was performed as previously described (Lauschke et al. 2020). Briefly, when hiPSC cultures reached near confluency, they were washed with DPBS and incubated with TrypLe™ for 1 min at 37 °C, after 1 min incubation TrypLe™ was carefully removed, and cells were observed under a microscope. The culture dish was further incubated at 37 °C for an additional 3 min to achieve a single-cell suspension. Cells were then resuspended in mTeSR-ROCK medium [mTeSR™ Plus containing 10 µM Y27632 dihydrochloride and 1% (V/V) PSG] and seeded into 96-well conical bottom plates at a cell density of 5000 cells/well. Following this, plates were centrifuged at 500 g for 5 min and incubated at 37 °C and 5%CO_2_ overnight. After 19 h (± 2h), EBs formed at the bottom of the wells. The medium was then replaced with Day 0 differentiation medium [KO-DMEM medium containing 10 µM Y27632, 1% (V/V) PSG, 0.1% (V/V) ITS, 10 ng/mL FGF2, 10 ng/mL Activin A, 2.5 µM CHIR99021 and 1 ng/mL BMP4]. After 24 h of incubation (Day 1), the medium was exchanged to TS medium [KnockOut -DMEM medium containing 1% (V/V) PSG, 40 nM sodium selenite, 5.5 µg/mL human transferrin and 25 µM Asc]. After another 24 h (Day 2), the medium was replaced with TS medium with addition of WNT. After 24 h (Day 3), the medium was changed back to TS medium and incubated for 72 h. On Day 6, the medium was refreshed with TS medium again. After the last 24 h, i.e., on Day 7, the cardiomyocytes differentiation was assessed.

### Cytotoxicity assessment

Cell viability after exposure to individual pyrethroids or 3-PBA was assessed as previously described (Lauschke et al. 2020) with some modifications. Briefly, near-confluent 2D hiPSC cultures were enzymatically dissociated into single cells using TrypLE™ and seeded at a density of 1 × 10^4^ cells per well onto Matrigel®-coated 96-well clear plates in mTeSR™ Plus medium supplemented with 1% (v/v) PSG and 10 μM Y27632 dihydrochloride. After allowing cells to adhere for 24 h, the medium was replaced with fresh mTeSR™ Plus containing 1% (v/v) PSG. Following an additional 24-h incubation, cells were treated with the test compounds at concentrations ranging from 0 to 100 μM in mTeSR™ Plus medium with 1% (v/v) PSG. This initial exposure lasted for 24 h, after which the medium was replaced with fresh medium containing the same test chemicals for a second 24-h period. After a total exposure time of 48 h, cell viability was measured using the CellTiter-Glo® Luminescent Cell Viability Assay (Promega, Wisconsin, USA). To perform the assay, 60 µL of the exposure medium was removed from each well, and 40 µL reagent was added. The solution was mixed thoroughly and incubated at room temperature for approximately 20 min. Luminescence was read using a PerkinElmer EnSpire 2300 Multimode Microplate Reader (PerkinElmer, Inc., Massachusetts, USA). For each compound, three independent experiments were performed in triplicates.

## Chemical exposure

### Single chemical exposure

Concentrated stock solutions of α-cypermethrin, deltamethrin, etofenprox and 3-PBA were prepared in dimethyl sulfoxide (DMSO) at 100 mM. Serial dilutions from the concentrated stocks were prepared in DMSO to obtain 1000 times the final exposure concentrations. For exposure, pyrethroid dilutions were added to the respective differentiation medium at a 1:1000 ratio on day 1, 2, 3 and 6 of cardiomyocyte differentiation, resulting in a final concentration of 0.1% DMSO across all wells. The concentration ranges were: 0 – 50 µM for deltamethrin, 0 – 25 µM for etofenprox, 0 – 100 µM for α-cypermethrin and 3-PBA.

A total of six independent experiments were conducted (n = 6). In three experiments, ten EBs were exposed per condition, while in the remaining three experiments, six EBs were exposed. The difference in EB numbers was due to different experimental design, the first three experiments were performed in a horizontal layout in 96‑well plates allowing ten EBs per condition, while the subsequent three experiments were performed in a vertical layout, allowing six EBs per condition and thereby enabling testing of a greater number of concentrations. Across all experiments, the total number of EBs per condition ranged between 45 and 48 due to occasional loss of EBs. For the lowest concentrations of the pyrethroids (α‑cypermethrin at 1.6 µM and 3.1 µM; etofenprox at 0.39 µM and 0.78 µM; deltamethrin at 0.78 µM and 1.6 µM) and 3‑PBA (3.1 µM), only three independent experiments were performed, resulting in 15–18 EBs per condition.

### Mixture exposure

Two fixed-ratio mixtures were prepared based on the compounds’ benchmark concentration (BMC) and lowest observed effect concentration (LOEC) values, which were derived from individual pyrethroids data in the PluriLum assay. The LOEC was defined as the lowest test concentration of the compound that caused an effect of sufficient statistical confidence (*p* value < 0.05). The same LOEC was determined statistically as 1.6 µM for each pyrethroid, resulting in an equimolar mixture with a total concentration of 4.8 µM at 1 × mixture concentration (LOEC mixture). The benchmark response level for the BMC determination was set at 10% inhibition according to statistical robustness criteria. The BMC_10_ values of the individual pyrethroids were estimated as 4.25 µM deltamethrin, 4.65 µM etofenprox and 1.64 µM α -cypermethrin, resulting in a total mixture concentration of 10.5 µM at 1 × mixture concentration (BMC_10_ mixture). The statistical BMC_10_ estimates for etofenprox was corrected to 2.70 µM after the mixture experiments, but since this value was not statistically different from the original 4.65 µM, we maintained the term “BMC_10_ mixture” for consistency. Both mixtures were tested across an enrichment range from 0.05 × to 7×. For each experimental condition, six EBs were exposed, and four independent experiments per mixture were conducted (n = 4).

### Quality control of embryonic bodies

On day 0, each EB was imaged using a BioTek Cytation 5 Cell Imaging Multimode Reader (Agilent Technologies, Santa Clara, USA) with a 4 × objective lens. Experiments were excluded if the average EB diameter fell below a predefined 500 µm threshold for quality control (Treschow et al. 2024b).

### PluriBeat assay – contractility of embryonic bodies

Beating activity of individual EB was evaluated on Day 7 using a light microscope (Nikon Eclipse Ts2, Tokyo, Japan). Each EB was observed for up to 15 s to assess its beating behaviour. Contractility was scored according to the following criteria: Full beat—the entire EB exhibited rhythmic contractions; Partial beat—only parts of the EB or localized areas showed contraction; No beat—no visible movement. These scores were used as a functional endpoint to evaluate the developmental effects of pyrethroids exposure relative to vehicle controls. For an experiment to be considered valid, at least 90% of vehicle control EBs on each plate were required to exhibit full beating on Day 7. The beat score data were analysed in R using a Proportional Odds Logistic Regression (POLR) framework, with beat scores treated as ordinal categories as previously described (Treschow et al. 2024b). For each concentration, we fitted the model

1 $$\mathrm{log}\left(\frac{P(Y>i}{P\left(Y<i\right)}\right)={\theta}_{i}+\beta x$$

where *x* is the compound concentration, *β* is the slope parameter, and ${\theta}_{i}$ is the intercept associated with beat scores greater than *i* (*i* =0,1). The response *Y* represents the cumulative log-odds of obtaining a beat score higher than category *i*. Each model included both control data and the respective concentration data. To test whether concentration affected beat scores, we compared the fitting model to a reduced model without the slope,

2 $$\mathrm{log}\left(\frac{P(Y>i}{P\left(Y<i\right)}\right)={\theta}_{i}$$

using a likelihood-ratio test. Model parameters were estimated with the ‘*polr*’ function from the ‘*MASS*’ package in R. Likelihood-ratio tests assumed that the difference in model deviances followed a chi-square distribution with 1 degree of freedom under the null hypothesis. *P*-values were obtained from the right tail of the distribution using the ‘*pchisq*’ function in R, with values < 0.05 considered statistically significant.

### PluriLum assay—*NKX2.5* expression

On day 7, *NKX2.5* gene activation was measured using the Nano-Glo® Luciferase Assay System (Promega, Wisconsin, USA), following the manufacturer’s protocol. EBs were washed with DPBS and transferred in 40 µL/well to a white 96-well plate, after which an equal volume of 40 U/mL papain was added to the wells. Plates were incubated for a minimum of 90 min at 37 °C, and EBs were dissociated by pipetting into single cell suspension. Half of the cell suspension was transferred to a new white 96-well plate containing 40 µL Nano-Glo® Luciferase Assay Substrate, and luminescence was measured after 5 min. The remaining half of the cell suspension was used to measure ATP levels with the CellTiter-Glo® Luminescent Cell Viability Assay (Promega, Wisconsin, USA). CellTiter-Glo® Substrate was added to the cell suspension and luminescence was measured after 20 min. For both assays, luminescence was measured using a PerkinElmer EnSpire 2300 Multimode Microplate Reader (PerkinElmer, Inc., Massachusetts, USA).

### RNA extraction

EBs were exposed to individual pyrethroids at their respective LOECs and to vehicle control. EBs were collected on Day 2 and Day 7 of cardiomyocyte differentiation. For each condition and time point, 18 EBs from three independent experiments were pooled to represent an average biological response and to ensure sufficient RNA yield for mRNA sequencing. Therefore, one pooled RNA sample was prepared per condition. RNA was extracted using a Qiagen RNeasy® Micro Kit (Qiagen, Germany) according to the manufacturer’s protocol, with a modified lysis step (75 µL buffer RLT instead of 350 µL to minimize guanidinium thiocyanate contamination). A solution of Buffer RLT containing 1% 2-mercaptoethanol (V/V) was added to the cell pellets, which were homogenized using a 20-gauge needle and a 1 mL syringe. Afterwards, 70% ethanol was added to the samples and mixed with the homogenized suspension. Samples were then transferred to RNeasy MinElute® spin columns. From this point, the RNA extraction protocol was followed according to the kit’s manual. RNA was eluted in RNase-free water and RNA concentrations were measured using a Nanodrop One Microvolume Spectrophotometer (ThermoFisher Scientific, USA). All extracted RNA samples had A260/A280 and A260/A230 ratios above 1.95. The RNA obtained was used for mRNA sequencing and gene expression analysis and was stored at -80°C until further analysis.

### RNA sequencing and gene expression analysis

Sequencing libraries and sequencing analysis were performed by Novogene Europe (Cambridge, UK). In brief, sequencing libraries were constructed by purification of total RNA using poly-T oligo-attached magnetic beads to obtain messenger RNA, followed by cDNA synthesis. Libraries were verified by fluorometric quantification and real-time PCR, followed by sequencing on an Illumina platform. Obtained data was cleaned and read counts were mapped to each gene. Differential gene expression analysis was performed using the *edgeR* package in R (Robinson et al. 2010) and the obtained p-values were adjusted using the Benjamini–Hochberg procedure to control the false discovery rate. Genes with a log_2_FoldChange ≥|1| and *p*-adjusted value < 0.05 were considered to be differentially expressed genes (DEGs). Enrichment analysis of Gene Ontology (GO) terms was performed using the *clusterProfiler* package in R (Yu et al. 2012). GO terms with an adjusted *p* value < 0.05 were considered significantly enriched terms. Venn diagram, and dot plot were prepared in R studio using the packages VennDiagram, ggplot2, and dplyr (Wickham 2007, 2014; Chen and Boutros 2011; R Core Team 2021).

To confirm the RNA sequencing results, qRT-PCR was performed for selected genes based on the determined GO terms, log_2_FoldChange and their involvement with embryonic development or cardiac function. cDNA was transcribed from 500 ng RNA using Omniscript® RT Kit (Qiagen, Germany), SUPERaseIn RNase Inhibitor (ThermoFisher Scientific, USA) and Random Primer Mix (New England Labs, USA). For RT-PCR, TaqMan Fast Universal PCR Master Mix (ThermoFisher Scientific, USA), TaqMan gene expression assays (ThermoFisher Scientific, USA) and 7.5 ng cDNA per reaction were used, on a QuantStudio 7 Flex Real-Time PCR System (ThermoFisher Scientific, USA). The primers used are presented in Table 1. *GAPDH* (glyceraldehyde-3-phosphate dehydrogenase) and *ACTB* (β-actin) were used as housekeeping genes.

**Table 1:** Primers used for RT-qPCR

Gene	Assay ID
*GAPDH*	Hs02786624_g1
*ACTB*	Hs01060665_g1
*KCNK3*	Hs00605529_m1
*FLT4*	Hs01047677_m1
*HMCN2*	Hs05015676_m1
*MMP25*	Hs00360861_m1

Each sample was measured in two independent experiments in duplicates, and relative gene expression was quantified using the 2^−∆∆Ct^ method (Livak and Schmittgen 2001). Samples with a cycle threshold (CT) difference between duplicates > 1 were excluded, and samples with CT values > 37 were considered non-detectable. Relative gene expression levels for each compound were compared to the negative control using an unpaired t-test in GraphPad Prism 10 (version 10.0.1). Data were tested for normality and log-normality using the Shapiro–Wilk test prior to analysis and are presented as mean ± standard deviation. Heterogeneity of variances across treatment groups was assessed using an F test. For HMCN2 expression measured by qRT‑PCR in EBs exposed to α‑cypermethrin, the assumption of equal variances was not met, therefore Welch’s t-test was applied.

### Concentration-effect regression analysis

For regression analysis, all concentration-effect data were normalized to the mean luminescence of the corresponding controls within each experiment. For statistical analysis, the mean of replicate-normalized values per experiment was used as the unit of analysis. Each individual pyrethroid and mixture was tested in six and four independent experiments, respectively, resulting in pooled datasets of six and four effect values per control and exposure condition. Median effect values from independent experiments were pooled, and exposure concentrations leading to more than 20% decreased cell viability was perceived as cytotoxic and excluded from statistical concentration–response analysis. As a non-random between-experimental variation was observed, with concentration-effect pattern slightly shifted on the log10(concentration) axis between experiments, the pooled data sets were described by a non-linear random effect regression model (Davidian and Giltinan 2003). Concentration-effect relationships were modelled according to the best-fit approach outlined by Scholze et al. (2001).

### Mixture modelling and analysis

The effects of the reconstituted mixtures were predicted according to the principle of concentration addition (CA) assuming non-interaction between the mixture compounds (Greco et al. 1995). For CA, the concentration of the mixture required to cause an *x*% inhibition (IC *x* mixture) was calculated based on the individual IC *x* values (IC *xi*) of each individual compound *i* that produces the same response *x*, and their relative molar contribution pi of each compound i to the mixture (Faust et al. 2003):

3 $${ICx}_{mixture}= {\left[\sum_{i=1}^{n}\left(\frac{{p}_{i}}{{ICx}_{i}}\right)\right]}^{-1}$$

To account for statistical uncertainty in the mixture predictions, a combined Monte-Carlo (MC) and nonlinear regression bootstrap simulation was conducted to establish approximate 95% confidence limits around the predicted mean response. For each compound, a distribution of resampled model fits was simulated using parametric bootstrapping, with resamples drawn from the fitted nonlinear effect model (Faust et al. 2003). These were then used as input for the MC simulation to produce a distribution of predicted mixture responses. Differences between predicted and observed mixture effects were deemed statistically significant when the 95% confidence intervals of the prediction did not overlap with those of the experimentally observed mixture effects.

## Results

### Effects of single pyrethroids on cardiomyocyte differentiation

Quality control confirmed that average EB sizes exceeded 500 µm on Day 0, and all EBs from the negative controls exhibited complete active beating on Day 7. Thus, all experiments were considered valid.

Cytotoxicity testing on undifferentiated hiPSC revealed that etofenprox reduced cell viability starting at 60 µM, whereas deltamethrin, α-cypermethrin and 3-PBA showed no cytotoxicity up to 100 µM (data not shown). Therefore, all test concentrations in the PluriBeat and PluriLum assay were below cytotoxic levels.

**Fig. 2:**
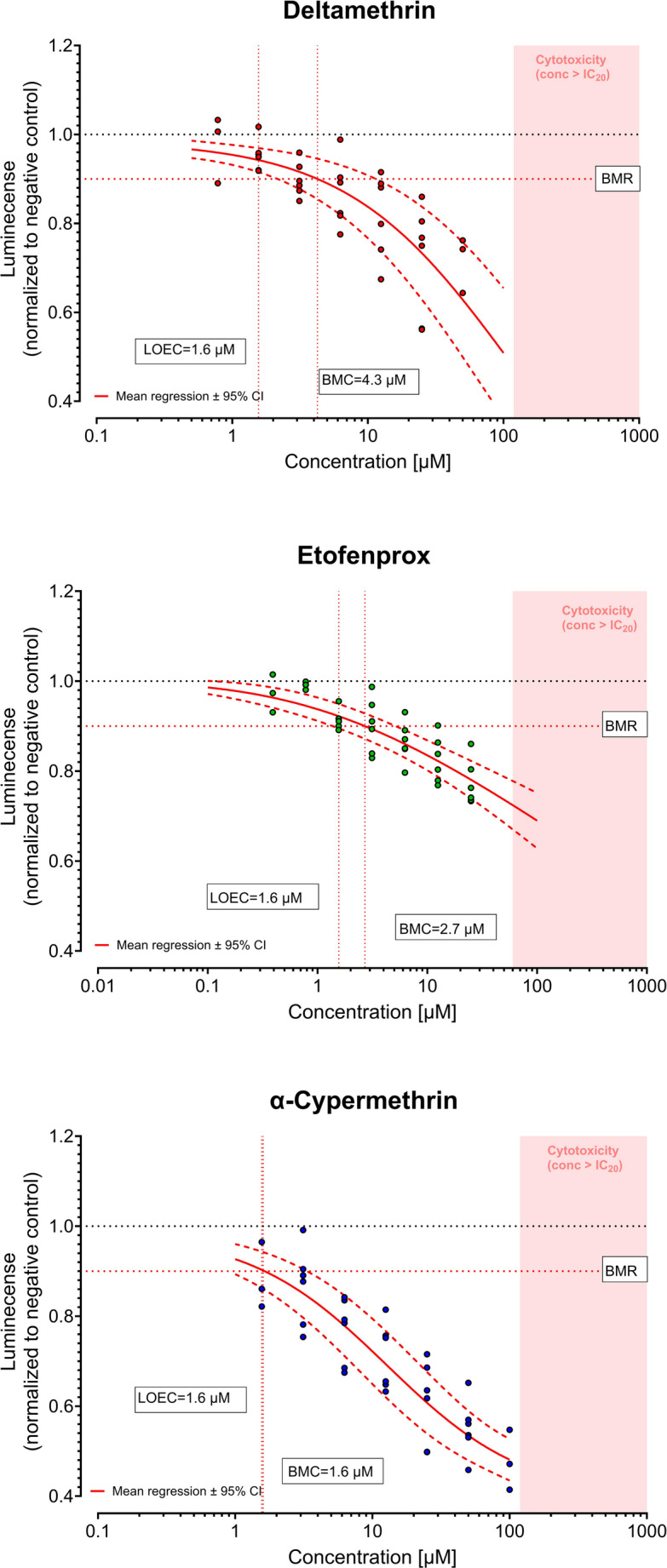
Concentration response data of deltamethrin, etofenprox and α-cypermethrin in the PluriLum assay. Data are shown as the replicate medians from six independent experiments (dots) and the mean ± 95% confidence intervals of the best-fit regression fit (solid and dashed lines). The 10% benchmark response (BMR) is indicated by a horizontal dotted line, the BMC₁₀ values are displayed along vertical lines extending from the point where each curve crosses the 10% BMR threshold. The lowest observed effect concentration (LOEC) is also included. Cytotoxic concentration range is highlighted by a pink area, with the concentration causing a 20% reduction in cell viability (IC_20_) chosen as threshold for cytotoxicity

In the PluriLum assay, all three pyrethroids induced a concentration-dependent decrease in luminescence (Fig. 2), reflecting reduced *NKX2.5* transcription and impaired cardiomyocytes differentiation. Deltamethrin exhibited a 10% inhibition of differentiation at 4.3 µM (BMC_10_), while BMC₁₀ values for etofenprox and α-cypermethrin were 2.7 µM and 1.6 µM, respectively. In contrast, 3-PBA showed no effect up to 100 µM (data not shown). For all three pyrethroids the LOEC was determined as 1.6 µM, with average effects of 8.5%, 4.7% and 11.8% observed for etofenprox, deltamethrin and α-cypermethrin, respectively.

**Fig. 3:**
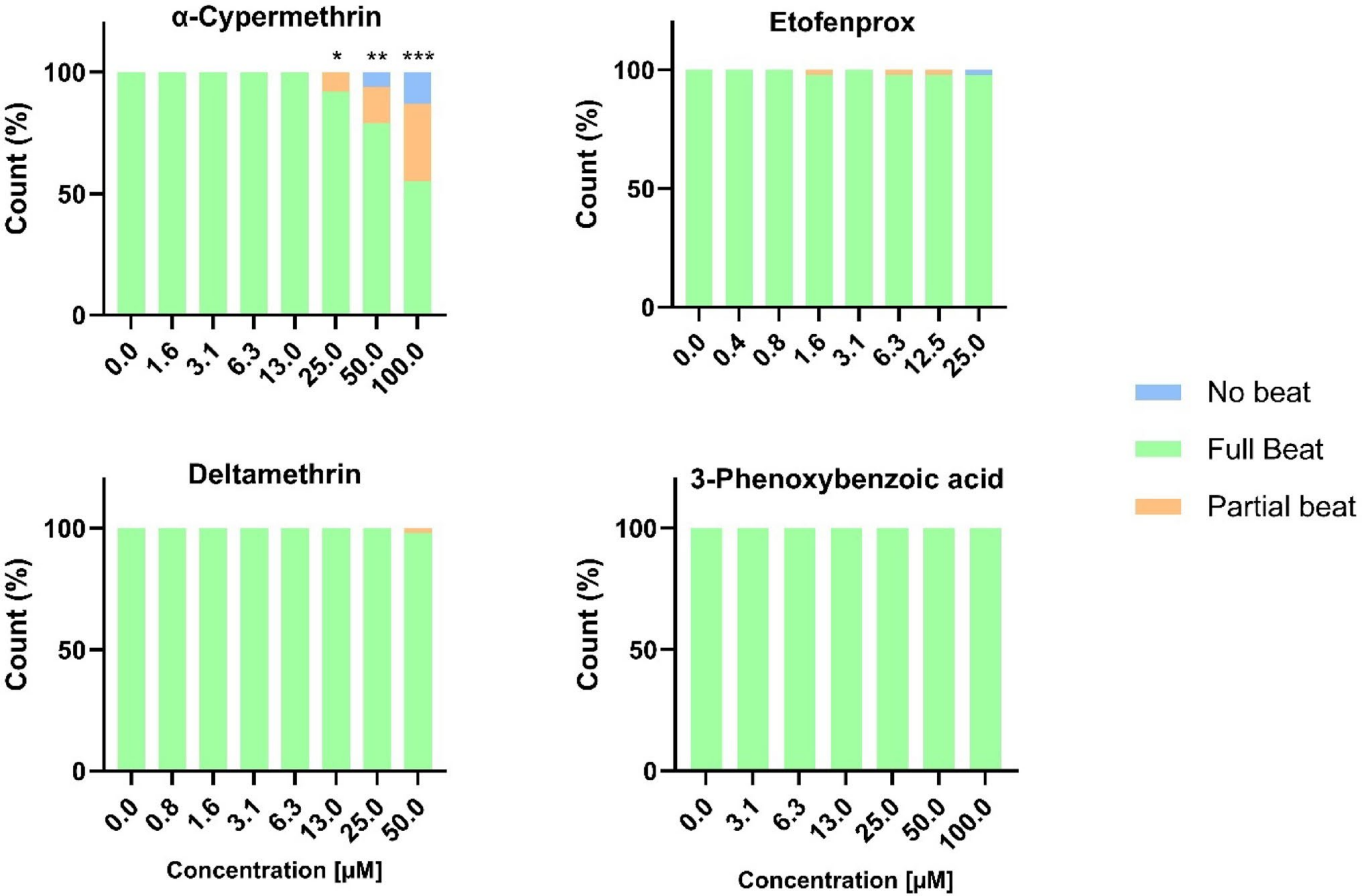
Effects on beat score in the PluriBeat assay of the three pyrethroids and 3-phenoxybenzoic acid. Statistical significance was assessed by proportional odds logistic regression. *p < 0.05, **p < 0.01, ***p < 0.001

In the less sensitive PluriBeat assay, α-cypermethrin impaired cardiomyocyte beating on Day 7 starting at 25 µM. At 100 µM, α-cypermethrin reduced the proportion of fully beating EBs to 55%, alongside 32% partially beating and 13% non-beating EBs (*p* < 0.05). In contrast, deltamethrin, etofenprox and 3-PBA had no significant effect on cardiomyocyte contractility on Day 7 at their highest tested concentrations (50 µM, 25 µM and 100 µM, respectively) (Fig. 3).

### Effects of pyrethroid mixture on cardiomyocyte differentiation

**Fig. 4:**
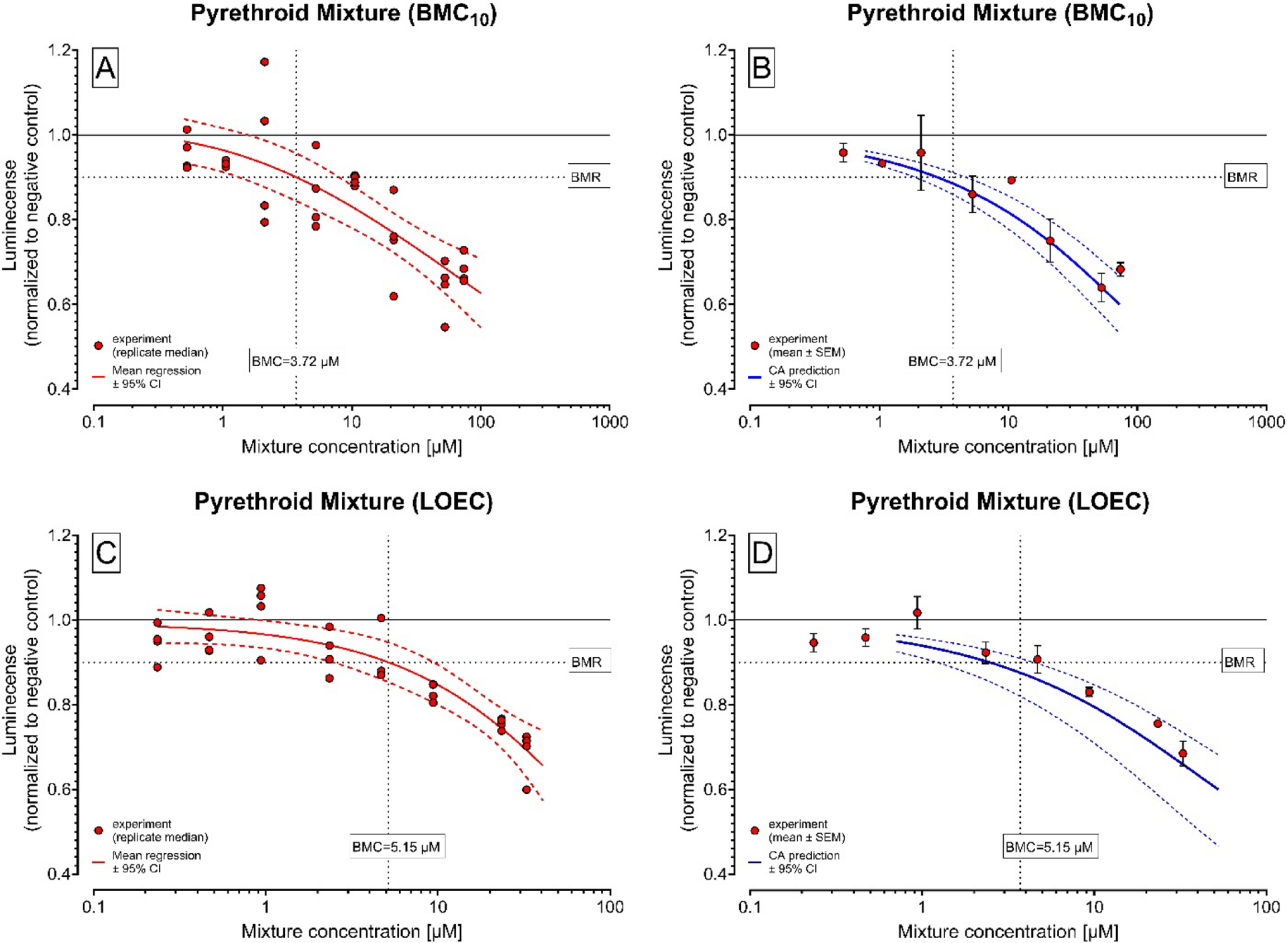
Experimental and predicted responses of the pyrethroid mixtures, composed of the BMC_10_ and LOEC values of the individual pyrethroids. (**A**) and (**C**): responses of the individual EBs (N = 6) and the nonlinear regression fit (mean ± 95% confidence interval, solid and dashed lines). (**B**) and (**D**): Prediction curve for additivity (mean ± 95% simulation interval, solid and dashed lines), together with experimental data (mean ± 95% SEM). The vertical dotted line represents the benchmark concentrations (BMC_10_) at 10% benchmark response (BMR)

Mixtures of pyrethroids did not show any indications of cytotoxicity across the tested concentration ranges (data not shown). Similarly, both mixtures did not affect cardiomyocyte beating on Day 7 across the tested concentration ranges (data not shown). In contrast, in the PluriLum assay, both mixtures inhibited cardiomyocyte differentiation in a clear concentration-effect pattern, with BMC_10_ values of 5.2 µM and 3.7 µM for the LOEC and BMC_10_ mixture, respectively (Fig. 4A and C). Regarding mixture effects, the experimentally observed effects aligned with the predictions based on CA over the entire response range, indicating that the pyrethroid combinations acted in a dose-additive manner (Fig. 4B and D).

### RNA sequencing

**Fig. 5:**
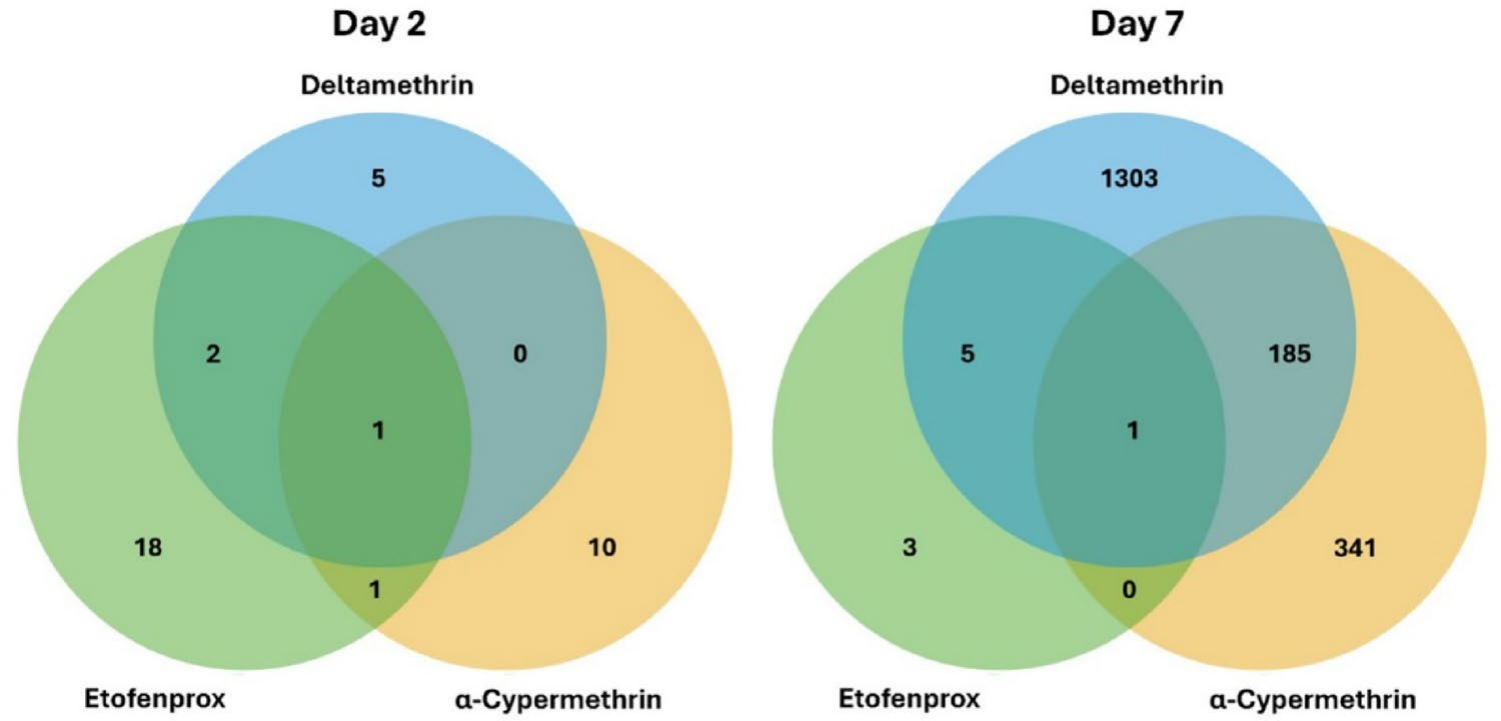
Venn diagram representing DEGs distribution among the evaluated pyrethroids at LOEC, on EBs collected at Day 2 and Day 7 of differentiation in the PluriLum assay. DEGs presented adjusted *p*-value < 0.05 and |Log2FoldChange|> 1

Transcriptomic changes following exposure to the three pyrethroids were evaluated in an exploratory RNA sequencing study. EBs exposed to the LOECs were collected on Day 2 and Day 7 of the cardiomyocyte differentiation protocol. On Day 2, we observed a total of 37 DEGs, of which 10 were unique to α-cypermethrin, 5 were unique to deltamethrin, and 18 were unique to etofenprox. On Day 7, we observed a total of 1838 DEGs, of which 341 were unique to α-cypermethrin, 1303 were unique to deltamethrin, and 3 were unique to etofenprox (Fig. 5). On both days, one gene was deregulated by all three pyrethroids: long intergenic non-coding RNA *FP236383.2* at Day 2, and long intergenic non-coding RNA *LINC02458* at Day 7. A list of DEGs is provided in the Supplementary Information.

**Fig. 6:**
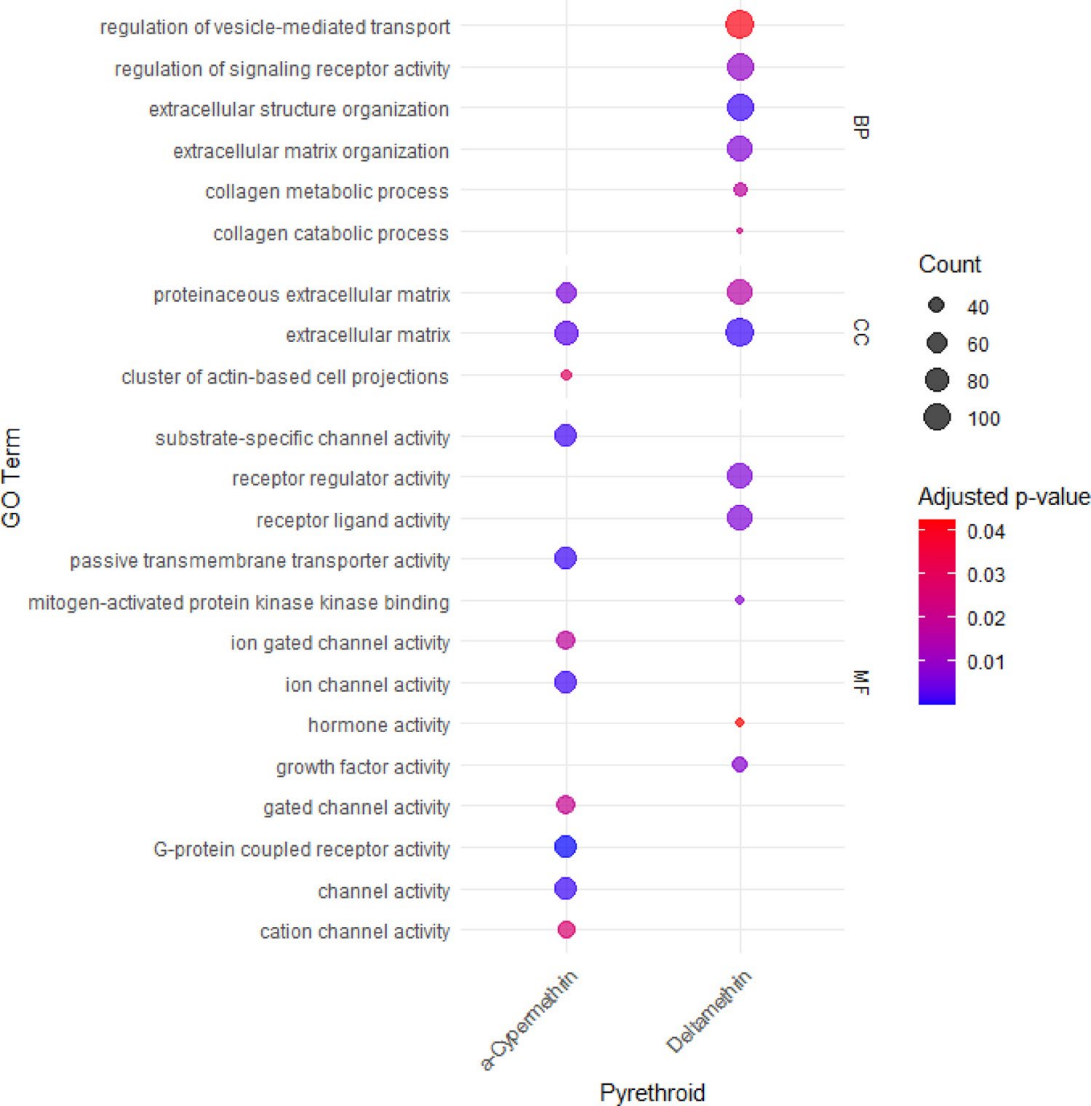
Representation of enriched GO terms in EBs collected on Day 7 following exposure to α-cypermethrin and deltamethrin in the PluriLum assay. The colour scheme represents the significancy of the adjusted p-values, while the dot size represents the number of genes involved in each GO term. BP: biological process, CC: cellular components, MF: molecular function

A GO enrichment analysis was performed to identify potential biological pathways affected by pyrethroid exposure on Day 7. Since substantially fewer DEGs were detected in EBs collected on Day 2, the GO terms from this time point were less informative, as all counts were below 25 (data not shown). Therefore, we only report GO enrichment results from the Day 7 samples. We observed that a total of 22 GO terms were significantly enriched, 11 GO terms for α-cypermethrin and 13 GO terms for deltamethrin (Fig. 6). The results showed that α-cypermethrin affected molecular functions (MF) related to channel activity, especially ion channels, while deltamethrin affected MFs related to receptor activity, and biological processes (BP) related to extracellular matrix organization and collagen processes. Both pyrethroids affected cellular components (CC) related to extracellular matrix. No GO terms were detected in etofenprox-treated Day 7 samples in the mRNA sequencing analysis. A list of all significantly enriched GO is presented in the Supplementary Information.

**Fig. 7:**
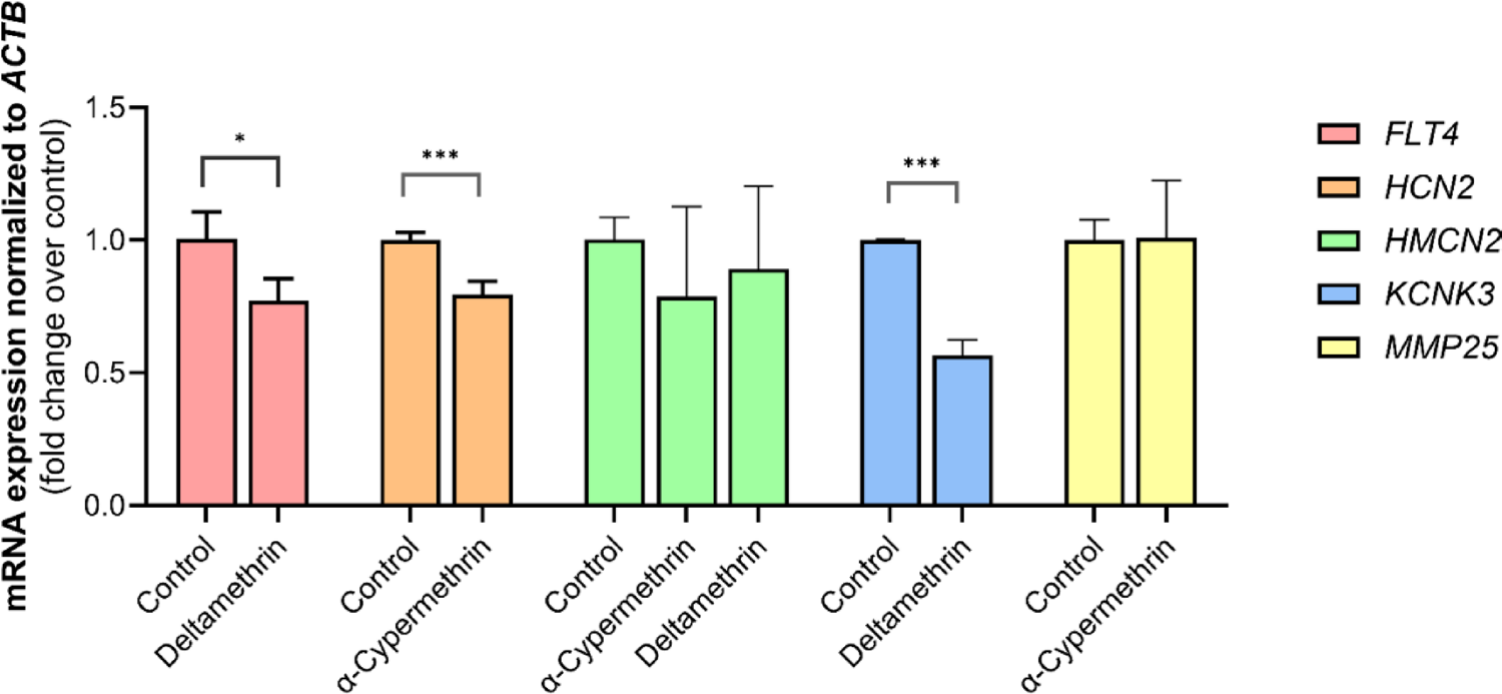
Validation of differentially expressed genes identified by mRNA sequencing using qRT-PCR. This figure shows fold change in mRNA expression of selected genes (*FLT4*, *HCN2*, *HMCN2*, *KCNK3*, and *MMP25*) normalized to *ACTB* in cardiomyocytes collected on day 7 following exposure to deltamethrin and α-cypermethrin. Expression levels are shown as fold change relative to the control group for each gene. The data are presented as mean ± SD (n = 4). *p ≤ 0.05, ***p < 0.001

Using RT-qPCR, we were able to validate the expression of most selected DEGs identified in the mRNA sequencing analysis of Day 7 samples (Table 2). As shown in Fig. 7, and in line with the mRNA sequencing results, the expression of *FLT4* and *KCNK3*, genes related to cardiac development and function, respectively, was significantly decreased by deltamethrin. On the other hand, α-cypermethrin exposure led to a reduction in the expression of *HCN2*, a gene that plays an important role in cardiac pacemaker activity. *HMCN2* expression was reduced by both deltamethrin and α-cypermethrin, although not significantly. *MMP25* expression did not change following α-cypermethrin exposure, in contrast to the results from mRNA sequencing.

**Table 2:** RT-qPCR validation of selected genes from mRNA sequencing analysis

Pyrethroid	Gene	Log2FoldChange
Deltamethrin	*FLT4*	− 2.08
	*HMCN2*	− 2.68
	*KCNK3*	− 2.17
α-Cypermethrin	*HCN2*	− 7.37
	*HMCN2*	− 2.78
	*MMP25*	− 7.31

## Discussion

The use of hiPSC-derived cells has emerged as a powerful approach for developmental toxicity testing of pesticides and other environmental chemicals. This is attributed to their strong human relevance, the reduced ethical concerns compared to animal experiments, and the scalability of in vitro systems. Several studies have demonstrated the utility of hiPSC-based assays for investigating developmental neurotoxicity of pyrethroids (Sirenko et al. 2019; Bartmann et al. 2023; Ishibashi et al. 2023) and of other chemical classes (Sirenko et al. 2019; Wages et al. 2020; Bartmann et al. 2023; Ishibashi et al. 2023; Wu et al. 2024).

Our results showed that deltamethrin inhibited cardiomyocyte differentiation with a BMC_10_ of 4.3 µM. Previous studies support the notion that deltamethrin can interfere with cardiac electrophysiological activity. In isolated adult cat ventricular myocytes, deltamethrin prolonged action potential duration and altered the kinetics of cardiac sodium channels at concentrations of 1 µM and 10 µM, respectively (De la Cerda et al. 2002). In an in vivo study in pregnant mice, exposure to deltamethrin at 3 mg/kg body weight increased embryonic mortality and reduced embryo viability. Embryonic cardiomyocytes from exposed litters exhibited slowed depolarization rates, prolonged action potentials, and shifted diastolic potentials (Luo et al. 2019). Similarly, in zebrafish embryos, 0.025 mg/L (approx. 0.05 µM) deltamethrin significantly reduced the expression of the voltage-gated sodium channel gene *Scn5lab* at 10 h post-fertilization, as well as the cardiac development-related genes *gata4* and *nkx2.5* at 24 h post-fertilization (Liu et al. 2021). In our study, deltamethrin did not impair cardiomyocyte contraction at concentrations up to 50 µM. In fact, Sirenko et al. (Sirenko et al. 2017) reported increased beat frequency at concentrations as low as 3 µM using hiPSC-derived cardiomyocytes in 2D monolayer cultures, with an assessment based on calcium flux measurements.

α-Cypermethrin and etofenprox also inhibited cardiomyocyte differentiation with BMC₁₀ estimates of 1.6 µM and 2.7 µM, respectively, based on the luminescence readouts. In vivo evidence supports potential cardiotoxicity of α-cypermethrin: a study on male Wistar rats reported that exposure to α-cypermethrin at 4 mg/kg body weight elevated cardiac oxidative stress and significantly increased plasma concentrations of cardiac markers (Ghazouani et al. 2020). In contrast, no studies to date have reported cardiac-specific effects following etofenprox exposure. Etofenprox is generally regarded as having low toxicity and is not classified as a teratogen (European Food Safety Authority 2009); however, our findings suggest that its potential to interfere with early human cardiac development warrants further investigation.

Both mixtures aligned well with the CA predictions, for both mixture ratios and across the entire response scale. This finding is consistent with other in vitro and in vivo studies of pyrethroid mixtures. An in vitro study using rat cortical neurons and glia exposed to a mixture of 5 pyrethroids (permethrin, deltamethrin, cypermethrin, β-cyfluthrin and esfenvalerate) showed additive effects on spontaneous neuronal network activity (Johnstone et al. 2017). Similarly, exposure of embryonic mouse cerebrocortical neurons to an equimolar mixture of 11 pyrethroids resulted in additive effects (Cao et al. 2011), in agreement with in vivo findings of dose addition using equipotent ratios of the same 11 compounds (Wolansky et al. 2009). Another in vivo study of adult male rats exposed to the same five-pyrethroid mixture as Johnstone et al. further supported the additive model for pyrethroid effects on motor activity, and indicated that differences in neurotoxicity among individual pyrethroids are driven mainly by toxicodynamic rather than toxicokinetic differences (Starr et al. 2012). It is important to note that other types of mixture effects, such as synergism, are rare and typically occur at higher exposure concentrations (Cedergreen 2014; Boberg et al. 2019). Therefore, considering the cumulative effect of co-occurring chemicals using standard models such as CA, is a critical component of chemical mixture risk assessment, while acknowledging that some chemical groups may present a higher risk of synergistic effects (Cedergreen 2014).

To gain insight into molecular alterations induced by pyrethroids during cardiomyocyte differentiation, we collected EBs exposed to pyrethroids at their LOECs derived from decreased *NKX2.5* expression in the PluriLum assay from two time points: Day 2 and Day 7. Comparing the differences in the amount of DEGs affected between Day 2 and Day 7, our data indicates that early-stage exposures induce subtle molecular changes that progressively expand into more pronounced and wide-ranging changes during later stages of cardiomyocyte differentiation. Only one gene was commonly deregulated by all three compounds at each time point, both non-coding genes, suggesting mostly distinct sites of action for the three pyrethroids but the same overall mode of action investigated in this study. GO analysis on EBs collected on Day 7 revealed that α-cypermethrin primarily disrupted MFs related to ion channel activity which is critical for cardiomyocyte contractility. Deltamethrin predominantly affects receptor activity and BPs associated with extracellular matrix organization and collagen synthesis, which structurally supports cardiac cells and play an essential role in cardiac development and remodelling (Rienks et al. 2014). Both pyrethroids also affected CCs related to the extracellular matrix, reinforcing potential impact on cardiac architecture by directly influencing genes or proteins associated with the extracellular matrix.

Notably, these changes were observed at LOECs derived from the PluriLum assay, highlighting that molecular effects which are associated with adverse developmental outcomes may occur at relatively low exposure levels. Future studies should explore a wider range of concentrations and different timepoints of exposure to better characterize the progression of pyrethroid-induced embryo-cardiac toxicity.

Finally, it should be acknowledged that RNA sequencing was conducted using only one biological replicate per treatment condition. As a result, the list of DEGs and enriched GO terms may change with the inclusion of additional replicates. Conesa et al. (2016) emphasize that, in the absence of replication, no formal statistical inference can be made and associated *p*-value should be interpreted with caution, limiting such analyses to an exploratory scope. Therefore, the transcriptomic results presented here provide preliminary insights into pyrethroid-induced transcriptomic changes, and further studies incorporating multiple biological replicates will be important to minimize batch effects and increase statistical power.

## Conclusion

This study investigated the developmental cardiotoxicity of three pyrethroids (deltamethrin, α-cypermethrin and etofenprox) as well as their shared metabolite 3-PBA using a hiPSC-derived cardiomyocyte differentiation model. The results showed that all three pyrethroids impaired cardiomyocyte differentiation, with a potency ranking of α-cypermethrin > etofenprox > deltamethrin, whereas the shared metabolite 3-PBA exhibited no effect. Mixtures of the pyrethroids also inhibited cardiomyocyte differentiation and followed the CA principle. A preliminary transcriptomic analysis revealed time-dependent and compound-specific gene expression changes, particularly in pathways related to ion channel activity and extracellular matrix organization, but with little overlap of genes between compounds. These findings highlight distinct molecular targets but a shared mode of action for the investigated pyrethroids and highlight the value of human-relevant in vitro models for evaluating early cardiac developmental toxicity.

## Supplementary Information

Below is the link to the electronic supplementary material.

Supplementary Material 1

## Data Availability

The datasets generated during and/or analysed during the current study are available from the corresponding author on reasonable request.
